# Flavonoid Profiles in the Pulp of Different Lemon Cultivars and Their Antioxidant Activity Based on UPLC–Q–TOF–MS

**DOI:** 10.3390/molecules29153464

**Published:** 2024-07-24

**Authors:** Zhixiang Liu, Peng Wang, Chengcheng Liu, Xin Tang

**Affiliations:** 1 Institute of Herbgenomics, Chengdu University of Traditional Chinese Medicine, Chengdu 611137, China; l18674128836@163.com; 2 Department of Pharmacy, Chengdu University of Traditional Chinese Medicine, Chengdu 611137, China; wangdapeng19981026@163.com; 3 Chongqing Key Laboratory of the Innovative Chinese Materia Medica & Health Intervention, Chongqing Academy of Chinese Materia Medica, Chongqing College of Traditional Chinese Medicine, Chongqing 400065, China; tangxin@cqctcm.edu.cn

**Keywords:** lemon, different cultivated varieties, antioxidant activity, flavonoids, UPLC–Q–TOF–MS

## Abstract

Previous studies have indicated that there may be differences among the varieties of lemon flavonoids, but the details have not yet been made clear, which limits the comprehensive use of different cultivated lemon varieties. In this study, ultra-performance liquid chromatography–quadrupole–time-of-flight–mass spectrometry (UPLC–Q–TOF–MS) and ultraviolet–visible spectroscopy (UV–Vis) were used to investigate the types and contents of flavonoids in the flesh of the main cultivated variety (Eureka) and five common lemon varieties, as well as their in vitro antioxidant activity. A total of 21 compounds were identified, five of which were common compounds. Among them, Verna, Lisbon, and Bearss each have characteristic components that can serve as potential criteria for variety identification. Each of the six varieties of lemon has strong antioxidant activity. The antioxidant activity of different lemon varieties is related to flavonoids. Therefore, Eureka and the other five varieties of lemon are good natural antioxidants, and the cultivation and industrial production of lemons should consider the needs and selection of suitable varieties.

## 1. Introduction

Lemon (*Citrus limon*) is one of the main cultivated citrus plants in the world; it is grown in more than 60 countries and regions (FAO Statistics, 2021) and is popular among consumers for its unique flavour [1,2]. Previous studies have shown that lemons likely originated in China or India from the three ancestors of the citrus genus, namely C. Reticulata, pomelo, and citron, that were hybridized [2,3,4]. Then, various locally cultivated varieties (e.g., Eureka, Lisbon, Femminello, and Fino) were produced after continued human intervention. These lemon varieties are important for industrial production. In addition to being used as fruits, lemons and their extracts are also processed for the production of other commodities, such as juice/juice beverages, preserved fruits, lemon essential oils, and food supplements. Recent studies have shown that lemons are rich in polyphenolic compounds, including flavonoids, phenolic acids, limonoids, and terpenoids, which have antioxidant, anti-inflammatory, antitumour, and lipid-lowering properties. They are widely used in fields such as medicine, food, and cosmetics [5,6,7,8,9,10]. However, research on different lemon varieties is limited, which leads to confusion in the cultivation and industrial production of lemon varieties. Currently, Eureka is the most commonly cultivated variety, and more research and understanding are needed to fully utilize different lemon varieties.

Flavonoids are the main phenolics in citrus plants and are the main compounds that exert biological activity [11,12]. Flavonoids that have been isolated and identified from lemons include eriodictyl, hesperidin, hesperetin, naringin, apigenin, diosmin, quercetin, lipocitrin, spinacetin, and neohesperidin. These components have been shown to be associated with lemon’s anti-inflammatory, antioxidant, antibacterial, antitumour, and lipid-lowering properties [5,6,8,10,11,12]. However, current research has focused on the chemical composition and pharmacological effects of individual lemon varieties, or on the composition of essential oils from the peels of multiple varieties of lemon. There have been no studies on the distribution of flavonoids in the flesh of different varieties of lemon fruit. Therefore, it is necessary to further study the composition and content of flavonoids in different varieties of lemon pulp.

Flavonoids play an important role in antioxidant activity and provide many of the health benefits of healthy food. The biological activity, composition, and content of flavonoids are closely related [13,14,15]. Previous studies have shown that lemon peel extract and lemon essential oil have good antioxidant activity [6,7,13]. The differences in the antioxidant activities of flavonoids in different varieties of lemon pulp are still unclear.

The aim of this study was to clarify the differences in flavonoid compounds and antioxidant activities in different varieties of lemon pulp. The composition and content of flavonoids in lemon pulp were measured using five representative lemon varieties and one main cultivated variety (Eureka). Then, the differences in antioxidant activity were compared, and the correlations between different flavonoids and antioxidant activity were analysed.

## 2. Results and Discussion

### 2.1. Total Phenolic Content

The detection results of TPC in different varieties of lemon pulp are shown in Table 1. The results showed that the total phenolic content in the lemon pulp ranged from 27.60 to 46.19 mg GAE/g DW, and the total phenolic content of the main cultivated variety (Eureka) was 35.59 mg GAE/g DW. The total phenolic content in the Fino lemon was the highest, followed by that in Lisbon, Femminello, and Verna lemons, all of which were significantly greater than the total phenolic content in Eureka and Bearss lemons (*p* < 0.05). This may be related to the extraction method and testing variety of phenolic compounds [14,16,17]. Compared with the distribution of phenolic compounds in different parts of lemon fruit [18], our results support that lemon pulp, especially that of Fino and Lisbon lemons, is a good source of phenolic compounds.

### 2.2. Total Flavonoid Content

The detection results of TFC in different varieties of lemon pulp are shown in Table 1. The results showed that the total flavonoid content in the lemon pulp ranged from 2.52 to 5.27 mg RE/g DW, and the total flavonoid content in the main cultivated variety (Eureka) was 3.74 mg RE/g DW. The TFC in the Fino lemon was the highest, followed by that in Femminello, Lisbon, and Eureka lemons, all of which were significantly greater than the TFC in Verna and Bearss lemons (*p* < 0.05). This may be related to the distribution of flavonoids in citrus fruits and the tested varieties [5,8,17,19,20,21]. Flavonoids are most abundant in the peel of citrus fruits and have a lower content in the flesh. Our results indicate that the composition of flavonoids strongly depends on the lemon variety, and further analysis is needed to determine the differences in the types and contents of flavonoids.

### 2.3. Flavonoid Compositions and Contents

Metabolomics is widely used in fields such as botany, medicine, food, and traditional herbal medicine due to its high information content, simple operation, universal metabolites, and overall reflection of metabolites in the body [15,22,23,24]. Metabolite profiling research, especially high-resolution mass spectrometry-based, highly selective metabolomics, has been widely applied in citrus research [23,25,26,27]. In this study, we used UPLC–Q–TOF–MS to determine the composition and content of flavonoids in different varieties of lemon pulp, and the results are represented by the relative content of each component (Table 2). A total of 21 compounds were detected, with 11, 10, 13, 11, 10, and 14 flavonoid components detected from Eureka, Verna, Fino, Femminello, Lisbon, and Bearss, respectively. There are five common components, namely hesperidin, narirutin, 5,7-dihydroxy-3′-methoxyflavone-4′-*O*-β-d-glucoside, (2*S*)-5,7-dihydroxy-6-methoxyflavanone-7-*O*-β-d-glucopyranoside., and swertiajaponin. Hesperidin and narirutin are the main flavonoid components of citrus fruits (pomelo, lime, sweet orange, citrus, and lemon), are closely related to lemon’s antioxidant, anti-inflammatory, and lipid-lowering properties, and are indicator components for the quality control of the traditional Chinese medicine Chenpi (Citri reticulatae pericarpium) [15,28,29,30]. The content of hesperidin in the different varieties of lemons ranged from 12.37% to 23.55%. Lisbon had the highest content, while Eureka had the lowest content. Similarly, the contents of narirutin in Eureka (2.68%), Verna (2.32%), and Fino (2.13%) lemons were significantly greater than those in Lisbon (0.20%), Femminello (0.20%), and Bearss (0.38%) lemons. In addition, Verna is the only cultivar with quercetin-3-gentiobioside-7-glucoside in quantifiable proportions. The unique components in the Lisbon lemon are 5,7,3′,4′-tetramethoxyflavones. There are five unique components in the Bearss lemon, including vicenin-II, luteolin-7,4′-*O*-β-d-diglucoside, violanthin, limocitrin, and nobiletin, but with relatively low contents. The differences in the composition and content of flavonoids in different varieties of lemon suggest that their potential biological activities also vary.

**Figure 1:**
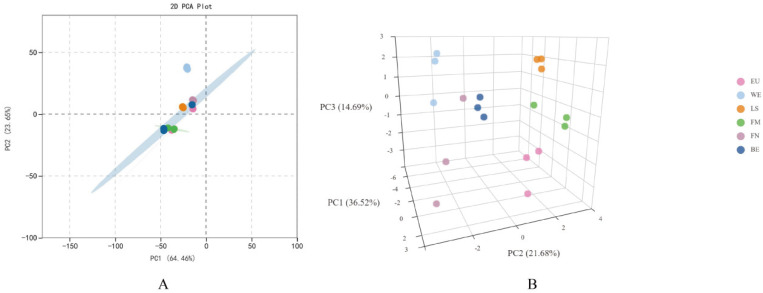
Principal component analysis results chart. (**A**) 2D PCA plot; (**B**) 3D PCA plot. EU: Eureka; WE: Verna; LS: Lisbon; FM: Femminello; FN: Fino; BE: Bearss.

**Figure 2:**
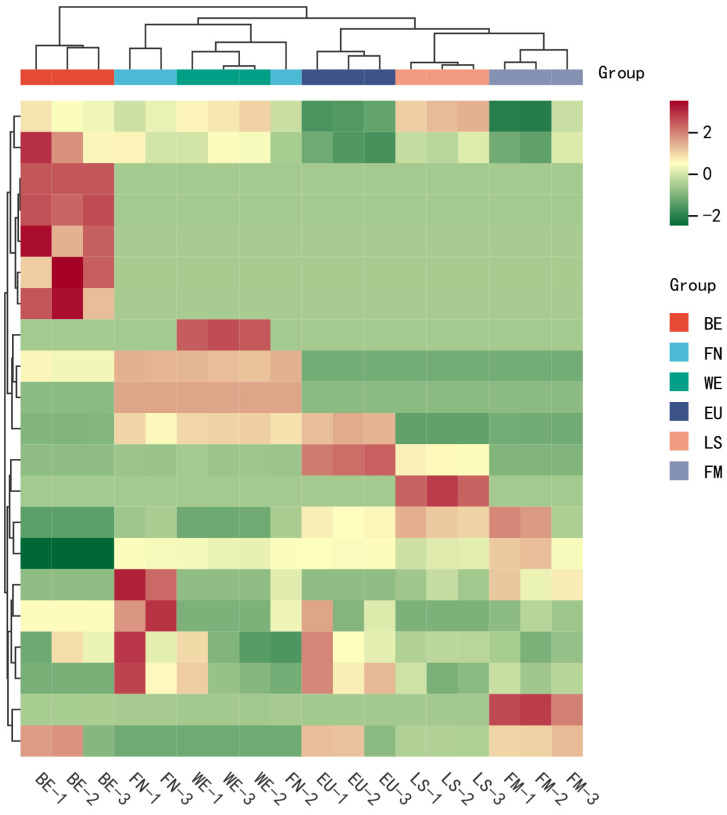
The cluster heatmap of flavonoids in different lemon varieties. BE: Bearss; FN: Fino; WE: Verna; EU: Eureka; LS: Lisbon; FM: Femminello.

Principal component analysis (PCA) of samples helps us understand the differences between group samples and the degree of variation within group samples, as shown in Figure 1. In the 3D PCA graph, samples from different groups are clustered separately, indicating that the metabolic profiles of different lemon varieties are different. The clustering of samples within the same group indicates a uniform distribution of metabolites, which confirms the repeatability and reliability of this experiment. According to the 2D PCA plot, Verna was significantly different from the five other lemons, indicating the uniqueness of its flavonoid compounds. To visualize the distribution of flavonoids in different varieties of lemon, we conducted a cluster heatmap analysis, as shown in Figure 2. Verna can be clustered separately from the other five types of lemons, consistent with the PCA results, and some flavonoid compounds have relatively high contents. The clustering of samples within the same group indicates that the composition and content of flavonoids in lemon pulp may be influenced by genotype.

### 2.4. Antioxidant Activity

**Figure 3:**
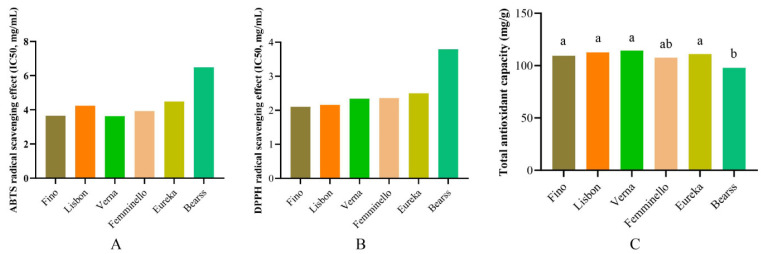
The antioxidant activity of the different lemon varieties. (**A**) ABTS measurement; (**B**) DPPH measurement; (**C**) The FRAP method was used to determine the total antioxidant activity of the different lemon varieties. The different lowercase letters indicate significant differences (*p* < 0.05).

The antioxidant activity of the six lemon varieties was evaluated using DPPH, ABTS, and FRAP, as shown in Figure 3. The results showed that the IC_50_ values of the DPPH and ABTS clearance rates of these lemons were 2.10–3.79 mg/mL and 3.62–6.49 mg/mL, respectively, with a total antioxidant capacity of 97.80–114.35 mg FeSO_4_/g DW, indicating differences in antioxidant activity among the different lemon varieties. The DPPH clearance rate of the Fino lemon was the highest, followed by those of the Lisbon, Verna, Femminello, and Eureka lemons, all of which were significantly greater than that of the Bearss lemon (*p* < 0.05). The ABTS clearance rate of the Verna lemon was the highest, followed by those of the Fino, Femminello, Lisbon, and Eureka lemons, all of which were significantly greater than that of the Bearss lemon (*p* < 0.05). The total antioxidant capacities of the Lisbon and Verna lemons were comparable, as were those of the Fino and Femminello lemons. The total antioxidant capacity of the Bearss lemon was significantly lower than that of the other five lemon varieties.

Our results indicate that the antioxidant activity reflected by different methods is not entirely consistent, which may be related to phenolic substances. There are differences in the types and contents of phenolic substances in different varieties of lemon pulp, resulting in different antioxidant activity detection results for different indicators [14,22]. Overall, the differences in antioxidant activity among the different varieties of lemon pulp were consistent with the trends in the total phenolic and flavonoid contents. The antioxidant activity of the different varieties of lemons is lower than that of oranges but greater than that of fruits such as pomelo, grapefruit, pear, watermelon, and mango [14,15,23].

### 2.5. Correlations between TPC, TFC, Flavonoids, and Antioxidant Activity

**Figure 4:**
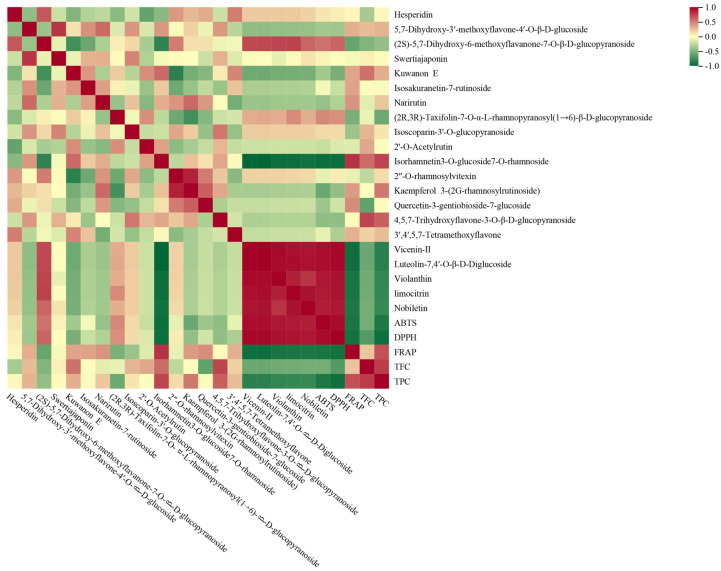
Pearson correlation analysis heatmap of flavonoids in different lemon varieties.

To investigate the correlation between flavonoids and antioxidant differences in different varieties of lemon, we conducted a Pearson correlation analysis, as shown in Figure 4. (2*S*)-5,7-Dihydroxy-6-methoxyflavanone-7-*O*-β-d-glucopyranoside, (2*R*,3*R*)-taxifolin-7-*O*-α-l-rhamnopyranosyl(1→6)-β-d-glucopyranoside, vicenin II, violanthin, limocitrin, nobiletin, and luteolin-7,4′-*O*-β-d-diglucoside were significantly positively correlated with the ABTS free radical scavenging rate, while isorhamnetin-3-*O*-glucoside-7-*O*-rhamnoside and kaempferol-3-(2G-rhamnosylrutinoside) were significantly negatively correlated with the ABTS free radical scavenging rate (|r| > 0.5, *p* < 0.05). (2*S*)-5,7-Dihydroxy-6-methoxyflavanone-7-*O*-β-d-glucopyranoside, vicenin II, violanthin, limocitrin, and luteolin-7,4′-*O*-β-d-diglucoside were significantly positively correlated with the DPPH free radical scavenging rate, and kuwanon E and isorhamnetin-3-*O*-glucoside-7-*O*-rhamnoside were significantly negatively correlated with the DPPH free radical scavenging rate.

Violanthin, limocitrin, nobiletin, luteolin-7,4′-*O*-β-d-diglucoside, vicenin II, (2*R*,3*R*)-taxifolin-7-*O*-α-l-rhamnopyranosyl(1→6)-β-d-glucopyranoside, and (2*S*)-5,7-dihydroxy-6-methoxyflavanone-7-*O*-β-d-glucopyranoside were significantly negatively correlated with FRAP. Interestingly, the TPC and TFC of the different varieties of lemon were significantly negatively correlated with the DPPH and ABTS free radical scavenging rates and significantly positively correlated with the FRAP rate. These results indicate that the composition and content of flavonoids in different varieties of lemons are different, which affects their antioxidant activity.

## 3. Materials and Methods

### 3.1. Plant Material

All lemon samples were collected from the planting base of the Lemon Industry Bureau in Anyue County, Sichuan Province, China, in November 2023. Four adjacent lemon trees of high yield and stable quality were randomly selected, and 6 fresh fruits of similar size that were free from pests and diseases were randomly selected from each tree. The varieties (Eureka, Lisbon, Femminello, Fino, Bearss, and Verna) were numbered and transported to the laboratory using dry ice. The samples were washed, the peel and pulp were manually separated, and the pulp was freeze-dried using the following method: The lemon pulp was evenly spread in a single layer and placed in a vacuum freeze dryer, cooled to −30 °C, and pre-frozen for 5 h before the heating plate temperature of the freeze dryer was set to 50 °C and the condenser temperature to −50 °C. After sealing, the pump was set to a pressure of 10 Pa and the sample dried for 72 h. The pulp was crushed with a grinder after drying, and all the samples were stored in a dry place at room temperature.

### 3.2. Chemicals

Methanol, sodium nitrite, aluminium nitrate trihydrate, sodium hydroxide, anhydrous sodium carbonate, anhydrous ethanol, potassium persulfate (analytical pure, Chengdu Cologne Chemical Co., Ltd., Chengdu, China). Rutin (chromatographically pure, Sichuan Vicki Biotechnology Co., Ltd., Chengdu, China). Gallic acid (chromatographically pure, Chengdu Ruifensidan Biotechnology Co., Ltd., Chengdu, China). Folin phenol reagent (1 mol/mL, Feijing Biotechnology Co., Ltd., Shanghai, China). 2,2-Diphenyl-1-Picrylhydrazyl (DPPH) and 2,2′-azino-bis(3-ethylbenzothiazoline-6-sulfonate) (ABTS) were both purchased from Shanghai Macklin Biochemical Technology Co., Ltd., Shanghai, China. Total antioxidant capacity (T-AOC) assay kit (ferric ion reducing antioxidant power, FRAP, Nanjing Jiancheng Biotechnology Research Institute, Nanjing, China).

### 3.3. Sample Preparation and Extraction

The sample processing followed the method of Ledesma Escobar et al. [31]. Lemon pulp powder (0.15 g) was accurately weighed, 5 mL of 80% methanol solution was added, and the mixture was mixed evenly. The sample was extracted using ultrasonication for 60 min (vortexing every 15 min). Then, the mixture was centrifuged for 15 min (4000 rpm/min), the supernatant was collected, and 0.22 μM filter membrane filtration was used. The filtrate was stored in an injection bottle for ultra-performance liquid chromatography–quadrupole–time-of-flight–mass spectrometry (UPLC–Q–TOF–MS) analysis.

### 3.4. Measurement of Total Phenolic Content

The Folin-Ciocalteu colorimetric method [32] was used to determine the total phenolic content (TPC), and the results are presented as mg gallic acid equivalents (GAE)/g dry weight (DW) of the pulp sample. Gallic acid solutions with concentrations of 0, 0.004, 0.005, 0.006, 0.008, 0.009, and 0.01 mg/mL were prepared. Then, 0.25 mL of phenol reagent, 4.75 mL of 20% sodium carbonate solution, 0.5 mL of gallic acid solution, or different concentrations of lemon extract were added, the solution was mixed well, 80% methanol was added to bring the volume to 15 mL, and the mixture was placed in a water bath at 75 °C for 30 min. The solution was cooled to room temperature, and the absorbance was measured at 760 nm. The standard curve was y = 10.697x − 0.0324 (R^2^ = 0.9994).

### 3.5. Measurement of Total Flavonoid Content

The total flavonoid content (TFC) was determined using the sodium nitrite–aluminium nitrate–sodium hydroxide colorimetric method [33]. A 0.2 mg/mL rutin solution consisting of 0 mL, 0.5 mL, 1.0 mL, 2 mL, 2.5 mL, 4.0 mL, 5.0 mL, or 3.0 mL of lemon extract was prepared. Then, the following solutions were added: 0.75 mL of 5% sodium nitrite, 0.75 mL of 10% aluminium nitrate, 6 mL of 4% sodium hydroxide, and, finally, 80% methanol to adjust the volume to 15 mL. After each addition, the mixture was mixed well and allowed to stand for 6 min, and for 15 min after methanol addition. The absorbance was measured at 510 nm. The standard curve was y = 12.55x − 0.0095 (R^2^ = 0.9998). The results are presented as mg rutin equivalent (RE)/g DW of the pulp sample.

### 3.6. UPLC–Q–TOF–MS Conditions

This study was analysed using the UPLC–Q–TOF–MS system (SynaptXS, Waters, Milford, MA, USA). The chromatographic conditions: (1) Column: ACQUITY UPLC CSH C_18_, 1.7 µm, 2.1 mm ×100 mm; (2) Mobile phase: A phase was ultrapure water, B phase was acetonitrile; (3) Elution gradient: 0~1.0 min 2% (B); 1.0~3.0 min 2% → 7% (B); 3.0~8.0 min 7% → 11% (B); 8.0~13.0 min 11% → 15% (B); 13.0~16.0 min 15% → 30% (B); 16.0~20.0 min 30% → 36%; 20.0~26.0 min, 36% → 70% (B); 26.0~30.0 min 70% → 85% (B); 30.0~31.0 min 85% → 95% (B); 31.0–33.0 min 95% → 2% (B); (4) Flow rate of 0.3 mL/min; Column temperature of 35 °C; Injection volume 2 μL.

Mass spectrum condition: positive and negative ion mode of electric spray ion source was adopted, capillary voltage: 3000 V; source temperature: 120 °C; desolvent gas temperature: 450 V; air curtain flow rate: 50.0 L/h; desolvent gas flow rate: 800.0 L/h; nebulizer gas flow rate: 6.0 Bar; low-end resolution: 4.7; high-end resolution: 15.0; IMS gas flow rate: 90.00 mL/min.

The collected raw data were imported into Compound Discoverer 3.0 software, the peak areas and peak alignments were extracted, and the measured spectra of secondary fragments were matched with the mzCloud network database and the local traditional Chinese medicine ingredient database (OTCML). The set filtering parameters for the matching results were as follows: peak area threshold value: 80,000, primary and secondary quality deviation: 5 ppm, and matching degree score: above 80. Compounds were analysed and identified by comparing the filtered ions with the compound information in the database.

### 3.7. Antioxidant Activity

DPPH. The DPPH assay was conducted following the protocol outlined by Wang et al. [15] with adjustments. The lemon extracts of different concentrations (100 μL) were combined with DPPH solution (0.008 mg/mL) (100 μL) in a 96-well plate. The mixtures underwent a 30-min reaction at room temperature in darkness, and the measurement of absorbance value at 517 nm was conducted using a microplate reader. In the control group, substituting the DPPH solution with 80% methanol, the blank group was similarly treated with 80% methanol. The IC_50_ value represents the DPPH clearance rate.

ABTS. Refer to Wang et al.’s method for ABTS determination, which was followed with slight modifications [15]. The K_2_S_2_O_8_ solution (2.6 mmol/L) was added to the ABTS solution (7.00 mmol/L) and allowed to react thoroughly in a cool place for 12–16 h. The solution obtained after dilution of the mixture seven times with 80% methanol (with an absorbance of 7.00 ± 0.02 at 734 nm) was used to prepare the ABTS working solution. Following that, 25 μL aliquots of lemon extract of different concentrations were introduced into the 96-well plate, the working solution of ABTS, a volume of 175 μL, was thoroughly mixed and allowed to react in darkness for a duration of 40 min, followed by measuring the absorbance value at 734 nm using a microplate reader; within the control group, substituting ABTS solution with 80% methanol, the blank group was also treated with 80% methanol. The IC_50_ value represents the ABTS clearance rate.

FRAP. The FRAP clearance rate was determined using a total antioxidant assay kit (Nanjing Jiancheng Bioengineering Research Institute, China). Overall, the standard determination curves of FeSO_4_ solutions with concentrations of 0.15, 0.3, 0.6, 0.9, 1.2, and 1.5 mmol/L were prepared. Then, 180 μL of FRAP working fluid was introduced into the wells of the 96-well plate, and 5 μL each of different concentrations of FeSO_4_ solutions and lemon extracts were added. This was followed by the addition of 5 μL of each solution in the blank group; the solution extraction was substituted with distilled water. Incubation of the mixture was carried out at 37 °C for 3–5 min, and the absorbance value was measured at 593 nm using a microplate reader. The standard curve is: y = 0.007x − 0.0067, R^2^ = 0.9974. The results are presented as mg FeSO_4_/g DW of the pulp sample. The standard curve is: y = 0.007x − 0.0067, R^2^ = 0.9974.

### 3.8. Statistical Analysis

The statistical function prcomp in R version 3.3.1 for principal component analysis, and the ComplexHeatmap package in R version 3.3.1 for hierarchical cluster analysis (HCA) and production of a heatmap were used. GraphPad Prism 8.4.3 (GraphPad Software company, La Jolla, CA, USA) was used for statistical analysis, plotting bar charts, and ANOVA analysis (*p* < 0.05 indicated statistical significance). All results are the average of three experiments.

## 4. Conclusions

This study evaluated the differences in TPC, TFC, and antioxidant activity among different lemon varieties. Fino had the highest total phenolic content, followed by Lisbon, Femminello, and Eureka, all of which were significantly greater than those of Verna and Bearss. Fino lemon had the highest total flavonoid content, followed by Femminello, Lisbon, and Eureka, all of which were significantly greater than those of Verna and Bearss. Fino had the highest DPPH clearance rate, followed by Lisbon, Verna, Femminello, and Eureka, all of which were significantly greater than that of Bearss. The ABTS clearance rate of Verna was the highest, followed by those of Fino, Femminello, Lisbon, and Eureka, all of which were significantly greater than that of Bearss. The total antioxidant capacities of Lisbon and Verna were comparable, as were those of Fino and Femminello. The total antioxidant capacity of Bearss was significantly lower than that of the other five lemon varieties. Furthermore, UPLC–Q–TOF–MS was used to determine the composition and content of flavonoids in different varieties of lemon pulp. A total of 21 compounds were detected, five of which were common components, such as hesperidin, narirutin, and swertiajaponin. Among them, Verna, Lisbon, and Bearss each had characteristic components that can serve as potential criteria for variety identification. The Pearson correlation analysis results indicate that vicenin-II, luteolin-7,4′-*O*-β-d-diglucoside, violanthin, limocitrin, and nobiletin are associated with antioxidant activity. Overall, our research revealed that Eureka and five other types of lemon are good natural antioxidants. There are differences in the composition and content of flavonoids in the different varieties of lemon, and the cultivation and industrial production of lemons should consider the needs and selection of suitable varieties.

## Figures and Tables

**Table 1:** The TPC and TFC in different varieties of lemon.

Sample	TPC (mg GAE/g DW)	TFC (mg RE/g DW)
FN	46.19 ± 1.08 a	5.27 ± 0.23 a
LS	41.06 ± 0.92 b	4.27 ± 0.11 c
WE	37.76 ± 1.25 c	2.43 ± 0.06 e
FM	37.91 ± 0.72 c	4.66 ± 0.03 b
EU	35.59 ± 0.77 d	3.74 ± 0.34 d
BE	27.60 ± 0.17 e	2.52 ± 0.06 e

Note: The different lowercase letters indicate significant differences (*p* < 0.05). FN: Fino; LS: Lisbon; WE: Verna; FM: Femminello; EU: Eureka; BE: Bearss.

**Table 2:** Volatile organic compounds identified in dry peel of Zangju at different ripening stages, sampled by GC–IMS.

No.	Compounds	Formula	tR (min)	Ion Adduction	Parent Ion (*m*/*z*)	Error (ppm)	Fragmentation Profile (*m*/*z*)	Relative Content (%)					
								EU	WE	FN	FM	LS	BE
1	Hesperidin	C_28_H_34_O_15_	11.50	[M − H]^−^	609.1825	0.0	593.1551, 301.0720, 286.0489	12.37	21.76	18.46	12.66	23.55	20.55
2	5,7-Dihydroxy-3′-methoxyflavone-4′-*O*-β-d-glucoside	C_22_H_22_O_11_	10.53	[M − H]^−^	461.1100	2.3	371.0802, 298.0491, 195.0671	1.12	0.44	0.85	0.36	0.22	0.03
3	(2*S*)-5,7-Dihydroxy-6-methoxyflavanone-7-*O*-β-d-glucopyranoside	C_22_H_24_O_10_	10.35	[M + H]^+^	463.1240	1.1	427.1048, 343.0831, 303.0523	1.70	4.92	4.42	2.89	4.13	7.57
4	Swertiajaponin	C_22_H_22_O_11_	11.49	[M + H]^+^	449.1442	0.0	301.0716, 263.0569, 153.0192	0.98	0.46	0.85	0.36	0.52	0.65
5	Kuwanon E	C_25_H_28_O_6_	9.33	[M − H]^−^	639.1592	3.9	595.1668, 287.0562, 151.0032	5.06	0.55	2.18	6.14	6.54	-
6	(2*R*,3*R*)-Taxifolin-7-*O*-α-L-rhamnopyranosyl(1→6)-β-d-glucopyranoside	C_27_H_32_O_16_	6.37	[M − H]^−^	625.1783	1.4	317.0676, 289.0737, 125.0239	26.30	-	-	34.64	11.41	30.64
7	Isosakuranetin-7-rutinoside	C_28_H_34_O_14_	17.56	[M − H]^−^	469.1880	2.5	229.1245, 227.1443, 179.0361	5.12	0.60	0.51	-	2.53	0.26
8	Narirutin	C_27_H_32_O_14_	13.53	[M − H]^−^	593.1891	2.5	559.1505, 519.1178, 285.0776	2.68	2.32	2.13	0.20	0.20	0.38
9	Isoscoparin-3′-*O*-glucopyranoside	C_28_H_32_O_16_	4.28	[M − H]^−^	611.1625	1.3	303.0533, 285.0419, 125.0208	0.51	-	1.07	0.17	-	0.62
10	2′-*O*-Acetylrutin	C_29_H_32_O_17_	6.62	[M + H]^+^	625.1787	3.9	487.1239, 355.0835, 289.0741	0.04	-	0.11	-	-	0.26
11	Isorhamnetin-3-*O*-glucoside-7-*O*-rhamnoside	C_28_H_32_O_16_	7.03	[M − H]^−^	623.1616	−0.3	503.1213, 413.0893, 383.0782	9.34	8.73	9.18	10.74	8.01	-
12	2″-*O*-rhamnosylvitexin	C_27_H_32_O_15_	12.31	[M − H]^−^	651.1584	2.7	579.1382, 375.0734, 360.0497	-	8.73	9.18	10.74	-	5.68
13	Kaempferol-3-(2G-rhamnosylrutinoside)	C_33_H_40_O_19_	9.97	[M − H]^−^	623.1638	3.3	593.1512, 285.0405, 283.0266	-	0.01	0.01	0.02	-	-
14	Quercetin-3-gentiobioside-7-glucoside	C_33_H_40_O_22_	9.31	[M − H]^−^	595.1666	−0.3	459.1161, 287.0564, 151.0034	-	35.72	-	-	-	-
15	Luteolin-7,4′-*O*-β-d-Diglucoside	C_27_H_30_O_16_	4.29	[M − H]^−^	341.1044	3.8	285.0427, 134.0379, 125.0217	-	-	-	-	-	0.05
16	3′,4′,5,7-Tetramethoxyflavone	C_19_H_18_O_6_	1.72	[M − H]^−^	833.1964	−3.5	391.0342, 217.0168, 87.0093	-	-	-	-	11.41	-
17	Vicenin-II	C_27_H_30_O_15_	9.32	[M + H]^+^	435.1290	0.9	289.07160, 153.0197, 135.0453	-	-	-	-	-	0.38
18	4,5,7-Trihydroxyflavone-3-*O*-β-d-glucopyranoside	C_21_H_22_O_10_	6.88	[M − H]^−^	785.2182	4.6	577.1583, 431.1019, 269.0467	-	-	2.67	1.53	0.35	-
19	Violanthin	C_27_H_30_O_14_	6.06	[M − H]^−^	593.1519	1.2	473.1112, 383.0791, 353.0680	-	-	-	-	-	0.99
20	Limocitrin	C_17_H_14_O_8_	10.28	[M − H]^−^	609.1474	2.1	461.1112, 341.0680, 298.0489	-	-	-	-	-	0.79
21	Nobiletin	C_21_H_22_O_8_	11.21	[M + H]^+^	579.1711	0.4	433.1141, 271.0605, 85.0304	-	-	-	-	-	0.39

## Data Availability

Data are contained within the article materials.
